# Arachidonic and Linoleic Acid Derivatives Impact Oocyte ICSI Fertilization – A Prospective Analysis of Follicular Fluid and a Matched Oocyte in a ‘One Follicle – One Retrieved Oocyte – One Resulting Embryo’ Investigational Setting

**DOI:** 10.1371/journal.pone.0119087

**Published:** 2015-03-12

**Authors:** Przemysław Ciepiela, Tomasz Bączkowski, Arleta Drozd, Anna Kazienko, Ewa Stachowska, Rafał Kurzawa

**Affiliations:** 1 Department of Reproductive Medicine and Gynecology, Pomeranian Medical University, Szczecin-Police, Zachodniopomorskie, Poland; 2 VitroLive Fertility Clinic, Szczecin, Zachodniopomorskie, Poland; 3 Department of Biochemistry and Human Nutrition, Pomeranian Medical University, Szczecin, Zachodniopomorskie, Poland; Friedrich-Loeffler-Institute, GERMANY

## Abstract

**Objective:**

To evaluate human oocyte ability to undergo fertilization and subsequent preimplantation embryonic development in relation to a wide panel of follicular fluid (FF) arachidonic acid derivatives (AAD) and linoleic acid derivatives (LAD) of prospectively selected patients undergoing intracytoplasmic sperm injection (ICSI).

**Methodology:**

Study was designed as a two center (a university clinic and a private clinic) prospective study. 54 women of 181 consecutive couples undergoing ICSI were prospectively found to be eligible for analysis. 'One follicle – one retrieved oocyte – one resulting embryo' approach was used. Each individual follicle was aspirated independently and matched to an oocyte growing in this particular follicular milieu. FF samples were assessed for AAD and LAD by high-performance liquid chromatography; additionally, activity of secretory phospholipase A (sPLA_2_) was determined by enzyme-linked immunosorbent assay.

**Principal Findings:**

Increased activity of sPLA_2_ and significantly higher AAD and LAD levels were found in FF of oocytes that did not show two pronuclei or underwent degeneration after ICSI in comparison to oocytes with the appearance of two pronuclei. Receiver operating characteristics curve analysis identified acids with the highest sensitivity and specificity: 5oxo-hydroxyeicosatetraenoic, 16-hydroxyeicosatetraenoic, 9-hydroxyoctadecadieneoic and 12-hydroxyeicosatetraenoic. No significant differences between AAD and LAD related to embryo quality were found.

**Conclusions/Significance:**

Our study demonstrates for the first time that elevated concentrations of AAD and LAD in FF at the time of oocyte retrieval significantly decrease the ability of oocytes to form pronuclei after ICSI. This may serve as a new tool for non-invasive assessment of oocyte developmental capacity. However, levels of AAD and LAD are not associated with subsequent embryo quality or pregnancy rate, and therefore more studies are needed to determine their usefulness in human IVF procedure.

## Introduction

Although there are many known techniques available to evaluate embryo quality [1–4], in vitro fertilization (IVF) needs reliable biomarkers of oocyte and sperm competence [4–8]. The concept to fertilize one good quality oocyte with a good quality sperm could revolutionize IVF and might lead to more patient-friendly stimulation, fewer IVF dropouts and elimination of ovarian hyperstimulation syndrome (OHSS) without affecting pregnancy rates. Live observation of the gametes morphology has not so far been good enough to ensure the right choice of embryos for normal future development [9–11]. On the other hand, to avoid invasive techniques that could damage oocyte structure, a composition of follicular fluid (FF) has been investigated as a possible source of follicle biomarkers for oocyte quality [12, 13].

**Fig 1:**
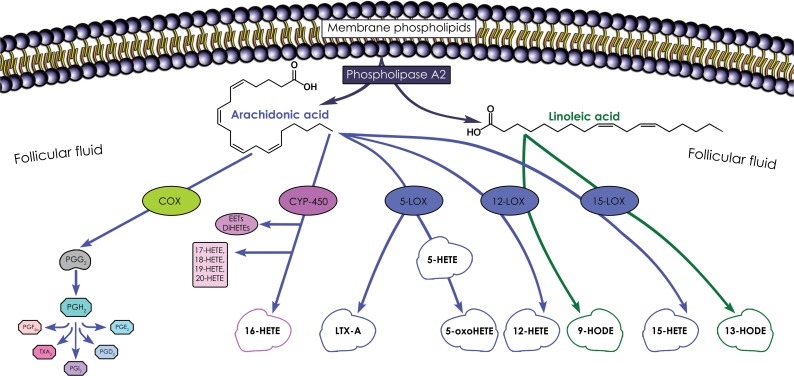
Follicular fluid arachidonic acid and linoleic acid pathways. Abbreviations: COX: cyclooxygenase; CYP-450: cytochrome P–450; DiHETEs: dihydroxyeicosatetraenoic acids; EETs: epoxyeicosatrienoic acids; HETE: hydroxyeicosatetraenoic acid; HODE: hydroxyoctadecadienoic acid; LOX—lipoxygenase, LTX: lipoxin, PG: prostaglandin; TX: thromboxane.

Dynamic ovarian follicular microenvironment reflects the secretory activity of granulosa and theca cells as well as the transfer of blood plasma constituents, responsible for the gradual acquisition of oocyte competence. There are two main polyunsaturated fatty acids (PUFA) present in human FF, which can potentially influence reproductive performance: arachidonic acid (AA) and linoleic acid (LA) [14, 15]. Both AA and LA are cleaved from phospholipids by phospholipases A_2_ (PLA_2_) (Fig. 1). Three major enzyme pathways metabolize AA: cyclooxygenase (COX), lipoxygenase (LOX) and cytochrome P450 monooxygenase (CYP), while LA is metabolized mainly through the LOX pathway [16]. Although the COX enzyme pathway has recently been given considerable attention [17, 18], little information is available on the possible importance of the LOX and CYP pathways [19, 20].

There are three active LOX family products in humans (5-lipoxygenase (5-LOX), 12-lipoxygenase (12-LOX), 15-lipoxygenase (15-LOX)) that metabolize AA to 5-, 12- and 15-hydroxyeicosatetraenoic (HETE) acids, respectively [5, 6]. Furthermore, 15-LOX converts LA to 13-hydroxyoctadecadienoic acid (13-HODE), whereas 12-LOX generates 9-hydroxyoctadecadienoic acid (9-HODE) from LA (Fig. 1). CYP pathway catalyzes formation of HETE acids from AA. The significance and specific role of HETE and HODE acids remains unknown [16].

Ovarian follicular fluid content together with the cumulus-oocyte complex is easily available during oocyte retrieval. Studies designed to determine predictor factors of oocyte quality normally consider pooled FF of all follicles and usually were not able to identify reliable markers of oocyte competence. In order to correlate FF substances with oocyte quality for study purposes, it is crucial that each individual follicle is aspirated independently and FF metabolites are matched to an oocyte growing in this particular follicular milieu. To make the results of our studies more decisive we used a ‘one follicle—one retrieved oocyte—one resulting embryo’ approach, which is more advanced in comparison to other studies, where only pooled follicular fluids from all follicles were used [21–24].

Therefore, the aim of this study was to carry out a prospective evaluation of a wide panel of follicular arachidonic acid (AAD) and linoleic acid derivatives (LAD) from selected patients undergoing ICSI and to correlate them with the oocyte ability to undergo fertilization and subsequent preimplantation embryonic development. Simultaneously, since the AA and LA release from a phospholipid molecule is controlled by phospholipases, the activity of secretory phospholipase (sPLA_2_) was measured.

## Materials and Methods

### Patients Selection Criteria

A study was conducted at the Department of Reproductive Medicine and Gynecology at Pomeranian University of Medicine and Private VitroLive Fertility Clinic in Szczecin. The ethics committee of Pomeranian Medical University, Szczecin, Poland, approved this study protocol—ethical authorization number: KB-0012/90/10. All patients signed for informed consent approved by the ethics committee of Pomeranian Medical University, Szczecin, Poland. The main aim of the study was to find a possible relationship between arachidonic acid derivatives and linoleic acid derivatives in follicular fluid and the matched oocyte ability to form pronuclei after intracytoplasmic sperm injection (ICSI) and to generate an embryo. According to study protocol women under the age of 38 years scheduled for controlled ovarian hyperstimulation (COH) and ICSI were considered eligible. Exclusion criteria included: 1) body mass index (BMI) ≥26 kg/m^2^; 2) meeting of 2003 Rotterdam polycystic ovary syndrome (PCOS) criteria; 3) moderate and severe male factor; 4) previous ineffective IVF/ICSI defined as none or <30% of oocyte fertilizations; 5) previous poor ovarian response to gonadotropin stimulation defined as ≤3 retrieved oocytes; 6) existence of ovarian cysts.

Standard GnRH agonist COH protocol with a starting dose of 150IU recombinant human FSH (Gonal F; Serono Pharma, Switzerland) was used. Final oocyte maturation was induced by a subcutaneous injection of 250 μg of alpha human choriogonadotropin (α-hCG) (Ovitrelle; Merck Serono, France) when the dominant follicle reached ≥18mm and the following two were ≥16mm.

### Follicular fluid collection

Follicular fluid was collected during routine oocyte retrieval (ROR) 36 h after α-hCG administration. Each ovarian follicle was aspirated independently and collected to a separate test-tube in order to identify matched single cumulus-oocyte-complex (COC). To obtain clean FF from a single follicle and avoid contamination from flush medium or mixed follicular fluid, FF samples were aspirated only from the first available follicle of every ovary. Test tubes with more than one COC were excluded from the study. In each case collected FF was checked afterwards for red blood cells. To remove cumulus cells a 10% hyaluronidase solution (Hyaluronidase Solution in Flushing Medium, FertiPro, Belgium) for less than 15 seconds was used. FF samples with matched M2 oocytes were centrifuged at 10,000 x g for 10 minutes, and the supernatants were aliquoted and stored at −80°C.

### Intracytoplasmic sperm injection protocol

During ICSI micro-manipulation holding pipette (K-HPIP-3335; Cook, USA) and injection pipette (K-MPIP-1035; Cook, USA) were used. Injected oocytes were placed in Sydney IVF Cleavage Medium G20720, K-SICM-20 (Cook Medical Inc., Bloomington, IN, USA) immediately after the procedure. This medium was used for the first 72 h of the culture and for the following 48 h Sydney IVF Blastocyst Medium G20722, K-SIBM-20 (Cook Medical Inc., Bloomington, IN, USA) was used. Post-fertilization embryo culture was carried out in single microdroplet (0.03 ml) of medium on Petri dish under a layer of mineral oil. Each fertilized oocyte was cultured in an individual drop.

### Embryological assessment

Embryos underwent conventional embryo assessment with standard checkpoints observed at fixed times: fertilization (observation of two pronuclei) between 16 and 18 h post ICSI; day 2 cleavage at 44 h (±1 h); day 3 cleavage (8 cells stage) at 68 h (±1 h); morula stage at 92 h (±2 h), and blastocyst stage at 116 h (±2 h). The evaluation of oocytes fertilization was done in agreement with Scott et al. criteria [25]. In this zygote grading system attention is paid to pronuclear size and symmetry, as well as size, number, equality and distribution of nucleoli. Scott et al. [25] classified zygotes into four groups (Z1-Z4) according to pronuclear morphology; where Z1 stands for equal pronuclei, equal number and size of nucleoli, aligned in both pronuclei at the pronuclear junction. In Z1 zygote the absolute number of nucleoli ranges between three and seven. In comparison, Z4 zygote has unequal or separated pronuclei with uneven size of nucleoli, randomly scattered in pronuclei. Embryos were evaluated according to the internal laboratory embryo grading system on the third day of the culture [26]. Embryo inspections were routinely performed in daily intervals depending on three morphological parameters: the number of cells, the appearance of blastomeres and the presence of cytoplasm defects or fragmentation. Class A embryos on the third day of the culture were defined as an embryo with ≥8 symmetrical, non-fragmented blastomeres. Blastocysts were evaluated on the fifth day of the culture according to grading system proposed by Gardner et al. [27] where three criteria are taken into consideration: appearance of trophectoderm, development of inner cell mass and expansion of the blastocyst cavity.

### Laboratory Analyses

Follicular fluid concentration of arachidonic acid derivatives (AAD): 5-HETE, 12-HETE, 15-HETE, 16(R)/16(S)-HETE and 5(S),6(R)-Lipoxin A4, 5(S),6(R), 15(R)-Lipoxin A4 as well as linoleic acid derivatives (LAD): 9-HODE, 13-HODE were assessed using high-performance liquid chromatography (HPLC) (S1 Fig.). AAD and LAD were extracted from the 0.5 ml of follicular fluid using solid-phase extraction RP-18 SPE columns (Agilent Technologies, UK) according to Raszeja-Wyszomirska et al. [28]. The HPLC separations were performed on Agilent Technologies 1260 liquid chromatography, consisting of model G1379B degasser, a model G1312B bin pump, a model G1316A column oven and a model G1315CDAD VL+. Samples were injected using a model G1329B. Agilent ChemStation software was used for instrument control and data acquisition and analysis. The separation was completed on a Thermo Scientific Hypersil BDS C18 column 100 x 4.6 mm 3 μm. The temperature of the column oven was set at 250°C. A gradient method was used, where the mobile phase was composed of a mixture of solvent A (methanol/water/acetic acid, 50/50/0.1, v/v/v) and B (methanol/water/acetic acid, 100/0/0.1, v/v/v). The per cent content of buffer B in the mobile phase was 30% at 0.0 min of separation, increased linearly to 80% at 20 min, was 98% between 20.1 and 23.9 min, and was 30% between 24 and 28 min [29]. The flow rate was 1.0 ml/min. The sample injection volume was 60 μl. The DAD detector monitored peaks by adsorption at 235 nm for 9-HODE, 13-HODE, 5-HETE, 12-HETE and 15-HETE, at 280 nm for PGB2 (Prostaglandin B2, internal standard) and 5oxo-ETE, at 210 nm for 16(R)/16(S)-HETE (the latter two were eluted as one peak) and at 302 nm for 5(S),6(R)-Lipoxin A4, 5(S),6(R), 15(R)-Lipoxin A4. Absorbance spectra of peaks were analyzed to confirm the identification of analytes. The quantization was based on peak areas with internal standard calibration.

Follicular fluid secretory phospholipase A_2_ (sPLA_2_) activity was assessed using enzyme-linked immunosorbent assay (ELISA). Cayman’s Chemical Company (AnnArbor, USA) sPLA_2_ Assay Kit and Diheptanoyl Thio-PC which served as a substrate for PLA_2_ with the Anti-sPLA_2_ (Human Type IIA) EIA Strip Plate was used. All procedures were performed according to the suggested assay protocol. Calculations of sPLA_2_ activity (μmol/min/ml per protein) were performed according to the manufacturer’s instructions.

### Statistical Analyses

The non-parametric Mann–Whitney test for comparisons between FF samples of fertilized and not fertilized, as well as fertilized and degenerated oocytes was used with p <0.05 considered as significant. Results are mostly expressed as mean ± standard deviation or percentages when appropriate. Receiver operating characteristics (ROC) curves were also fitted to obtain cut-off values for AAD, LAD using the area under the curve (AUC). Statistical analysis was conducted using Statistica 10 software (StatSoft Inc., USA).

## Results

The study flow is showed in S2 Fig. Out of 181 couples, scheduled for COH and ICSI between February and September 2012, 54 couples, a mean women’s age of 34.36±2.96 years and a mean BMI 23.5±0.9 kg/m^2^, were considered eligible. During 54 oocyte retrievals 70 FF samples according to ‘one follicle—one retrieved oocyte—one resulting embryo’ were qualified to study group. The mean diameter of aspirated follicles was 19.84±1.48 mm and average volume of collected follicular fluid was 3.58±1.55 ml.

The embryological and clinical outcome is presented in Table 1. 16–18 h after ICSI 40 oocytes (57.14%) revealed normal fertilization (2 pronuclei), 18 oocytes (25.17%) showed no fertilization and 12 oocytes (17.14%) underwent degeneration. 20 zygotes (50%) in studied group were evaluated as Z1, 13 zygotes (32.5%) as Z2 and further 7 zygotes (17.5%) as Z3 according to Scott et al. criteria [25]. 64–68 h following ICSI 18 class A embryos were transferred to the uterus on the third day of the culture. The following 11 class A and 4 class B embryos managed to develop to blastocysts and were vitrified. 8 pregnancies from the studied oocytes were achieved—six single and two twin pregnancies. Single pregnancies ended after 37th week of gestation and both twin pregnancies between the 35th and 36th week of gestation. It is important to emphasize that the results of the treatment in the studied group (high degeneration rate after ICSI: 17.14% or pregnancy rate: 47.05%) do not correspond to the actual overall effectiveness, because not all retrieved oocytes or developing embryos were included in the studied group.

**Table 1:** Embryological and clinical outcome of the studied group.

		M2		M1		GV	
		n	%	n	%	n	%
**Total collected COC**	n = 467	388/467	83.09%	**52/467**	11.13%	27/467	5.78%
**Studied group COC**	n = 70	70	-	-	-	-	-
		**Fertilization**		**Absence of fertilization**		**Oocyte degeneration**	
		**n**	%	**n**	%	**n**	%
**Total collected M2 oocytes**	n = 388	327/388	84.28%	40/388	10.31%	21/388	5.4%
**Studied group M2 oocytes**	n = 70	40/70	57.14%	18/70	25.71%	12/70	17.14%
		**Z1**		**Z2**		**Z3**	
		**n**	%	**n**	%	**n**	%
**Total zygotes** †	n = 327†	110/327	33.63%	98/327	29.97%	115/327	35.17%
**Studied group zygotes**	n = 40	20	50%	13	32.5%	7	17.5%
		**Class A**		**Class B**		**Class C**	
		**n**	%	**n**	%	**n**	%
**Total D3 embryos** ^#^	n = 287^#^	126/287	43.90%	148/287	51.56%	13/287	4.53%
**Studied group D3 embryos**	n = 40	29	72.5%	11	27.5%	-	-
		**Pregnancy rate**		**Single pregnancy**		**Twin pregnancy**	
		**n**	%	**n**	%	**n**	%
**Total D3 embryo transfers**	n = 54	20/54	37.03%	17/54	31.48%	3/54	5.55%
**Studied group D3 embryo transfers**	n = 18	8/17	47.05%	6/17	35.29%	2/17	11.76%
**Implantation rate**							
**D3 embryos transferred**	n = 101	23/101	22.77%				
**Studied group D3 embryos transferred**	n = 18	9/18	50%				
		**Blastocyst stage**		**Development arrest**			
		**n**	%	**n**	%		
**Total D5 embryos**	n = 186	69/186	37.10%	117/186	62.90%		
**Studied group D5 embryos**	n = 22	15/22	68.18%	7/22	31.82%		

Abbreviations: Studied group: ‘one follicle—one retrieved oocyte—one resulting embryo’ FF group; COC: cumulus-oocyte-complex; M2: mature metaphase II oocyte; M1: immature metaphase I oocyte; GV: germinal vesicle; Z1, 2, 3: zygote grading according to Scott et al. [25]

†: 4 zygotes assessed as Z4 not included; Class A, B, C: embryo grade according to internal laboratory embryo score standards on the third day of the culture [26]

#: 40 zygotes were arrested in development; pregnancy rate: defined as USG viable fetus; implantation rate: defined as percentage of implanted embryos compared to the number of transferred embryos; D3: third day of culture, D5: fifth day of culture.

**Fig 2:**
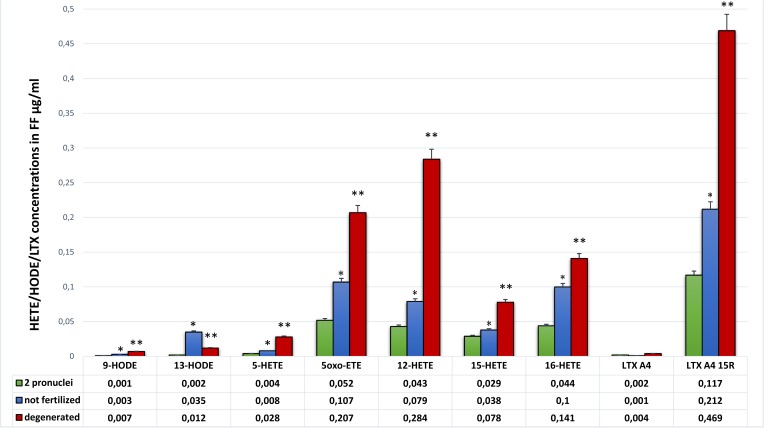
Comparison of HETE/HODE/LTX mean concentrations in follicular fluid of fertilized, unfertilized and degenerated oocytes after ICSI. U Mann Whitney: * p<0.001 for difference between fertilized and unfertilized oocytes; ** p<0.001—for difference between fertilized vs. degenerated oocytes. HETE: hydroxyeicosatetraenoic acid, HODE: hydroxyoctadecadienoic acid, LTX: lipoxin, FF: follicular fluid

We found significant differences in the HODE and HETE concentrations between FF of oocytes that after ICSI were fertilized, not fertilized or degenerated (Fig. 2). Mean concentrations of 9-HODE and 13-HODE were significantly lower in FF of fertilized oocytes in comparison to oocytes that did not form pronuclei or were not viable after ICSI (0.001 μg/ml vs. 0.003 μg/ml, p<0.004 and vs. 0.007 μg/ml, p<0.000 for 9-HODE; and 0.002 μg/ml vs. 0.035 μg/ml, p<0.001 and vs. 0.012 μg/ml, p<0.006 for 13-HODE, respectively). Mean concentrations of 5-HETE, 5oxo-ETE, 12-HETE, and 16-HETE in FF of fertilized oocytes were 2 times lower than in unfertilized ones (0.004 μg/ml vs. 0.008 μg/ml, p<0.009; 0.052 μg/ml vs. 0.107 μg/ml, p<0.001; 0.043 μg/ml vs. 0.079 μg/ml, p<0.003; 0.044 μg/ml vs. 0.100 μg/ml, p<0.009; respectively). The mean difference was even higher when FF of fertilized oocytes was compared to the concentrations of LAD and AAD from degenerated oocytes (Fig. 2).

Receiver operating characteristics (ROC) curve analysis showed the highest areas under the curve (AUCs) for 5oxo-ETE, 16-HETE, 9-HODE and 12-HETE (0.812, 0.766, 0.758 and 0.732, respectively), yielding 65% to 96% sensitivity and 54% to 93% specificity (Table 2, S3 Fig.). Concentrations of LAD, both lipoxins, 5oxo-ETE and 16-HETE were significantly lower in FF of oocytes which subsequently development after ICSI into zygotes that were assessed as optimal (Z1 and Z2) in comparison to suboptimal Z3 according to Scott et al [25]. Further ROC analysis of those derivatives showed that 5oxo-ETE had AUC as high as 0.816 with 83% sensitivity and 71% specificity (S1 Table). Although lipoxin A4 15R had the highest AUC, it revealed only 50% specificity.

**Table 2:** Receiver operating characteristic analysis of LAD, AAD and fertilization.

		Cut off point [μg/ml]	Sensitivity [%]	Specificity [%]	AUC	95% CI
**LAD**	**9-HODE**	0.003	96	54	0.758	(0.587–0.929)
	**13-HODE**	0.006	99	54	0.699	(0.503–0.896)
**AAD**	**5-HETE**	0.016	29	93	0.669	(0.489–0.849)
	**5oxo-ETE**	0.066	75	93	0.812	(0.665–0.958)
	**12-HETE**	0.062	65	77	0.732	(0.571–0.893)
	**15-HETE**	0.040	85	57	0.716	(0.590–0.926)
	**16-HETE**	0.048	64	85	0.766	(0.603–0.930)
	**LTX A4**	0.003	82	24	0.490	(0.299–0.682)
	**LTX A4 15R**	0.074	57	77	0.702	(0.535–0.869)

Abbreviations: LAD: linoleic acid derivatives, AAD: arachidonic acid derivatives, AUC: area under curve, HETE: hydroxyeicosatetraenoic acid, HODE: hydroxyoctadecadienoic acid, LTX: lipoxin

We did not find any statistical difference between LAD and AAD mean concentrations in relation to embryo class on the third (S2 Table) and fifth day (S3 Table) of the culture and to pregnancy rate (S4 Table).

Analysis of secretory phospholipase A_2_ showed significantly enhanced activity of sPLA_2_ in FF of oocytes that degenerated after ICSI or were not fertilized in comparison to normal fertilizations (1.427 and 1.256 vs. 0.886 μmol/min/ml per protein, p<0.002 and p<0.003, respectively).

## Discussion

To our knowledge, this is the first prospective study, to correlate specific panel of AAD and LAD in FF aspirated independently from individual follicles with the fate of matched oocytes. Whereas the majority of human studies published measured the COX/PGL pathway of arachidonate cascade, we have investigated products of LOX and CYP pathway gaining new data about their possible impact on oocyte competence. We have found significantly increased concentrations of HODEs and HETEs in the FF of oocytes that after ICSI were unable to form pronuclei or underwent degeneration in comparison to those which achieved fertilization. Obtained results showed that AAD and LAD correlated with the oocyte competence, however, not all derivatives correlated with zygote quality and neither AAD nor LAD demonstrated a relationship with embryo quality. Analysis of sPLA_2_ presented significantly enhanced activity in FF of oocytes that degenerated or were not fertilized after ICSI in comparison to normal fertilization. Combining obtained results together with sPLA_2_ activity we managed to find a possible explanation for AAD and LAD accumulation in FF with impaired oocytes. Our results suggest that the increase in sPLA_2_ activity, followed by amplified AAD and LAD synthesis, elicited probably by LOX and CYP pathways, at the time of oocyte retrieval, may be responsible for an oocyte inability to form pronuclei, regardless of meiotic maturation.

Several pieces of data from literature suggest that sPLA_2_ action affects cell death and survival [30]. Available studies demonstrate that sPLA_2_ specific binding to the motoneuronal cell surface, followed by internalization and enzymatic activity-dependent induction of apoptosis, possibly as a consequence of extensive extra- and intracellular AA release, is necessary for cell damage and apoptosis. Other sets of studies on neurodegenerative diseases showed that sPLA_2_ enzyme activity organizes a cascade of changes including not only inflammation but also cell degeneration [31]. Although the details of molecular sPLA_2_ induced apoptosis mechanism have not been fully clarified, there is much data showing various possibilities. Choi et al. revealed that sPLA_2_ promoted cell death by activating matrix metalloproteinase-9 and increasing type I collagen degradation [32]. Yu et al. showed that sPLA_2_ attenuates growth and promotes apoptosis predominantly via its effects on nuclear factor kappa-light-chain-enhancer (NF-κB) activity [33]. In addition, sPLA_2_ may initiate signaling in several other pathways that mediate cell survival including phosphatidylinositol 3-kinase-AKT (PI3K-AKT), extracellular-signal-regulated kinase (ERK) and p38 mitogen-activated protein kinase (p38 MAPK). The p38 MAPK is a member of the mitogen-activated protein kinase (MAPK) family that participates in a signaling cascade in response to cytokines and stress in somatic cells [34]. A study by Villa-Diaz et al. showed that in porcine oocyte maturation p38 MAPK becomes active around GVBD and has a role in M1-M2 transition, suggesting that p38 MAPK might be involved in the FSH-induced meiotic resumption of oocytes [34]. Based on these data we can hypothesize that, even though the MAPK pathway is necessary for meiotic maturation, uncontrolled sPLA_2_ activity can disturb critical cellular functions required for homeostasis and may decide upon oocyte reproductive competence [35].

The consequence of prolonged and increased sPLA_2_ activity is a substantial increase in AAD and LAD release which both have one common feature—they are strong neutrophil chemoattractants and inflammatory activators. Correspondingly, Smith et al. found a variation in leukocyte subtypes within FF, which cannot be solely accounted for blood vessel damage during oocyte retrieval [36]. Therefore, it can be postulated that a substantial participation of neutrophils in FF at the time of oocyte retrieval may alter the ability of oocyte to undergo fertilization. Kawano et al. reported that higher levels of monocyte chemoattractant proteins were associated with follicles bearing mature oocytes compared with those bearing immature oocytes [37]. Furthermore, it is well established that areas of inflammation are infiltrated in coordinated stages, and polymorphonuclear leukocytes are frequently first to arrive at inflammation site to be followed by monocytes/macrophages [38]. Thus, our results suggest that at the time of oocyte retrieval higher levels of chemoattractant AAD and LAD may disrupt oocyte competence.

Eicosanoid pathways, either through COX or LOX, add molecular oxygen that generates reactive oxygen species [39,40]. Depletion of the scavenging capacity of antioxidant defense systems is hazardous to cells and leads to oxidative damage. However, there is ongoing debate whether oxidative stress (OS) affects oocyte competence. In our study we have found strong negative correlation between high levels of both 9-HODE and 13-HODE and oocyte competence which are well known markers of OS. Preliminary results published by several authors showed OS impact on spindle organization disturbances in mouse oocytes [41–43] or disruption of fertilization in mature hamster oocytes [44]. Following clinical studies on human oocytes revealed confusing results [21–24, 45–49], mainly because of different materials (follicular fluid, culture medium or embryos), assay methods (lipid peroxidation—LPO, total antioxidant capacity—TAC, antioxidants and antioxidant enzymes) and the end points (presence of an oocyte, fertilization, embryo viability and pregnancy). Although no significant association was observed between oocyte maturation and follicular fluid reactive oxygen species [49], LPO and TAC levels [45, 47, 50], there was a significant negative correlation between glutathione peroxidase, reactive oxygen species, TAC and LPO values in follicular fluid and embryo quality [21, 23, 51].

Finally, in view of confusing data, a recent robust study by Fujimoto et al. evaluated the relevance of a vast panel of antioxidant enzyme activities and specific lipid peroxidation end products by correlational analyses with early embryo morphology parameters, embryo fragmentation, and embryo cell number, using a 1 follicle—1 oocyte/embryo design, analogous to our study [39]. Although the authors did not find correlations between lipid peroxidation derivatives or antioxidant enzyme activities and embryo quality, post hoc power analysis indicated possible undetected associations between the embryo fragmentation score and 13-hydroxy octadecatrienoic acid (13-HOTE) and 13-hydroperoxy octadecadieneoic acid (13-HPODE). Despite the fact that Fujimoto et al. did not consider the oocyte competence as an end point, this result suggests a possible detrimental association between 13-HOTE and 13-HPODE on embryo development. Although 9- and 13-HPODE are products of radical mediated enzymatic oxidation via lipoxygenase, they instantaneously become substrates of many enzymes such as glutathione peroxidases or phospholipases. Thus, the stable oxidation products available are HODEs [40].

It is difficult to study a biological variation of biomarkers inside the follicle of the healthy individual during natural cycle. Therefore, our knowledge is based on the broad series of experiments and studies performed mainly on other mammals. However, studies on human oocyte competence are possible during IVF. In order to correlate FF substances with oocyte quality for study purposes, it is crucial that each individual follicle is aspirated independently. Studies designed to determine predictor factors of oocyte quality typically considered pooled FF of all follicles and usually were not able to identify reliable markers of oocyte competence. Therefore, methodology of our study has been given careful consideration.

Firstly, to avoid the effect of maternal age on oocyte quality, and the potential negative impact of sperm quality on embryo development, we decided to exclude women older than 38 years of age and men with moderate or poor sperm parameter. Additionally, concentration of circulating free fatty acids increases with age and since follicular fluid is mainly plasma-derived, age was an important exclusion criterion [52]. Moreover, to avoid possible fat tissue accumulation bias or oxidative stress bias, patients with BMI ≥26 kg/m^2^ or PCOS were prospectively disqualified. Therefore, the substantial number of excluded patients made the study group highly cohesive.

Secondly, since we were able to draw conclusions only if each individual follicle was aspirated independently, we decided to include in the study FF only from the first aspirated follicle of both ovaries. Obviously, in this method AAD and LAD profiles were representative for the lead follicle, not the cohort of follicles. However, different approach would make oocyte retrieval difficult and dangerous for the patient due to multiple vaginal punctures and the numerous flushing of the needle with culture medium after every follicular puncture, as well as an increased risk of vaginal bleeding. This limitation resulted from safety reasons. Nevertheless, it is important to emphasize that we were aware of intra-follicular AAD/LAD variation in selected patients but we have obtained similar, therefore robust values for specific FF/oocyte profiles within studied group.

Thirdly, we decided to include couples scheduled for COH and ICSI only, as a denuded oocyte can be examined and its maturity can be assessed. Literature supports the idea that oocyte ICSI degeneration is operator-independent [53]. Nevertheless, we decided that in this study one skilled and fully trained scientist with over 7 years of experience performed ICSI exclusively. However, we were aware that there could be an unknown effect of ICSI upon the results. For example, it could be possible that elevated AA and LA derivatives signify oocytes that have compromised plasma membranes and perhaps would fertilize normally by insemination, but cannot stand the physical rigor of membrane breaching during ICSI. However, a control group of oocytes fertilized by standard insemination, included to see if failed pronuclear formation was related to elevated AADs and LADs, would not resolve this doubts, because we could not assess status of oocyte in cumulus-oocyte-complex before fertilization, whether it was mature or maybe already degenerated. Furthermore, during standard IVF there are important events that are taking place, such as sperm penetration through layers of supporting granulosa cells, sperm penetration and membrane fusion, which are not present in ICSI procedure. All mentioned stages might be AAD/LAD dependent or AAD/LAD independent. And our main goal was to assess intra-oocyte changes in ICSI procedure.

Unfortunately, we were unable to study more derivatives because of FF sample volume limitation. Therefore, LOX expression and activation as well as COX pathway products require further studies. Also, since humans do not synthesize LA and AA is mainly synthesized from dietary linoleic acid, a detailed evaluation of patients’ diet should be performed. Changes in eating habits between IVF cycles could explain the observed findings.

In conclusion, the presented results suggest that high levels of linoleic acid and arachidonic acid derivatives, probably due to enhanced activity of secretory phospholipase A2, significantly decrease human oocyte ability to be fertilized in ICSI cycles. They also raise questions about the role of LOX and CYP pathways during natural and stimulated cycles. Intriguingly, measurements of AAD and LAD may be a novel tool used in IVF for non-invasive assessment of oocyte developmental capacity. Nonetheless, it is important to emphasize that our results also indicate that levels of AAD and LAD are not associated with subsequent embryo quality or pregnancy rate, and therefore more studies are needed in this area to determine their usefulness in human IVF procedure.

## Supporting Information

S1 Fig Exemplary HPLC chromatogram with monitored peaks by adsorption at 235 nm (5-,12-, 15-HETE and 9-, 13-HODE) in follicular fluid. Abbreviations: HETE: hydroxyeicosatetraenoic acid, HODE: hydroxyoctadecadienoic acid,
(TIF)

S2 Fig Flow chart of the patients eligible for the study and the number of obtained FF samples. Abbreviations: PCOS: polycystic ovary syndrome; BMI: body mass index; COC: cumulus-*oocyte-complex*.
(TIF)

S3 Fig ROC analysis of HETE/HODE/LTX concentrations measured by HPLC in follicular fluid and fertilization. Abbreviations: ROC: receiver operating characteristic, HETE: hydroxyeicosatetraenoic acid; HODE: hydroxyoctadecadienoic acid; LTX: lipoxin.
(TIF)

S1 Table ROC analysis of HETE/HODE/LTX concentrations and zygote quality according to Scott et al. [25]. Abbreviations: ROC: receiver operating characteristic; HETE: hydroxyeicosatetraenoic acid; HODE: hydroxyoctadecadienoic acid; LTX: lipoxin; LAD: linoleic acid derivatives; AAD: arachidonic acid derivatives; AUC: area under curve.
(DOCX)

S2 Table Comparison of HETE/HODE/LTX concentrations in follicular fluid based on embryo quality on 3rd day of the culture. *U Mann Whitney; Abbreviations: LAD: linoleic acid derivatives; AAD: arachidonic acid derivatives; HETE: hydroxyeicosatetraenoic acid; HODE: hydroxyoctadecadienoic acid; LTX: lipoxin; SD: standard deviation.
(DOCX)

S3 Table Comparison of HETE/HODE/LTX concentrations in follicular fluid based on embryo quality on 5th day of the culture. *U Mann Whitney; Abbreviations: LAD: linoleic acid derivatives; AAD: arachidonic acid derivatives; HETE: hydroxyeicosatetraenoic acid; HODE: hydroxyoctadecadienoic acid; LTX: lipoxin; SD: standard deviation.
(DOCX)

S4 Table Comparison of HETE/HODE/LTX concentrations in follicular fluid of transferred embryos in relation to pregnancy. *U Mann Whitney; Abbreviations: LAD: linoleic acid derivatives; AAD: arachidonic acid derivatives; HETE: hydroxyeicosatetraenoic acid; HODE: hydroxyoctadecadienoic acid; LTX: lipoxin; SD: standard deviation.
(DOCX)
