# Supplementary figures and images for: Arachidonic and Linoleic Acid Derivatives Impact Oocyte ICSI Fertilization – A Prospective Analysis of Follicular Fluid and a Matched Oocyte in a ‘One Follicle – One Retrieved Oocyte – One Resulting Embryo’ Investigational Setting
Source: PLoS One. 2015 Mar 12;10(3):e0119087. doi: 10.1371/journal.pone.0119087 (PMC4357448; doi:10.1371/journal.pone.0119087)

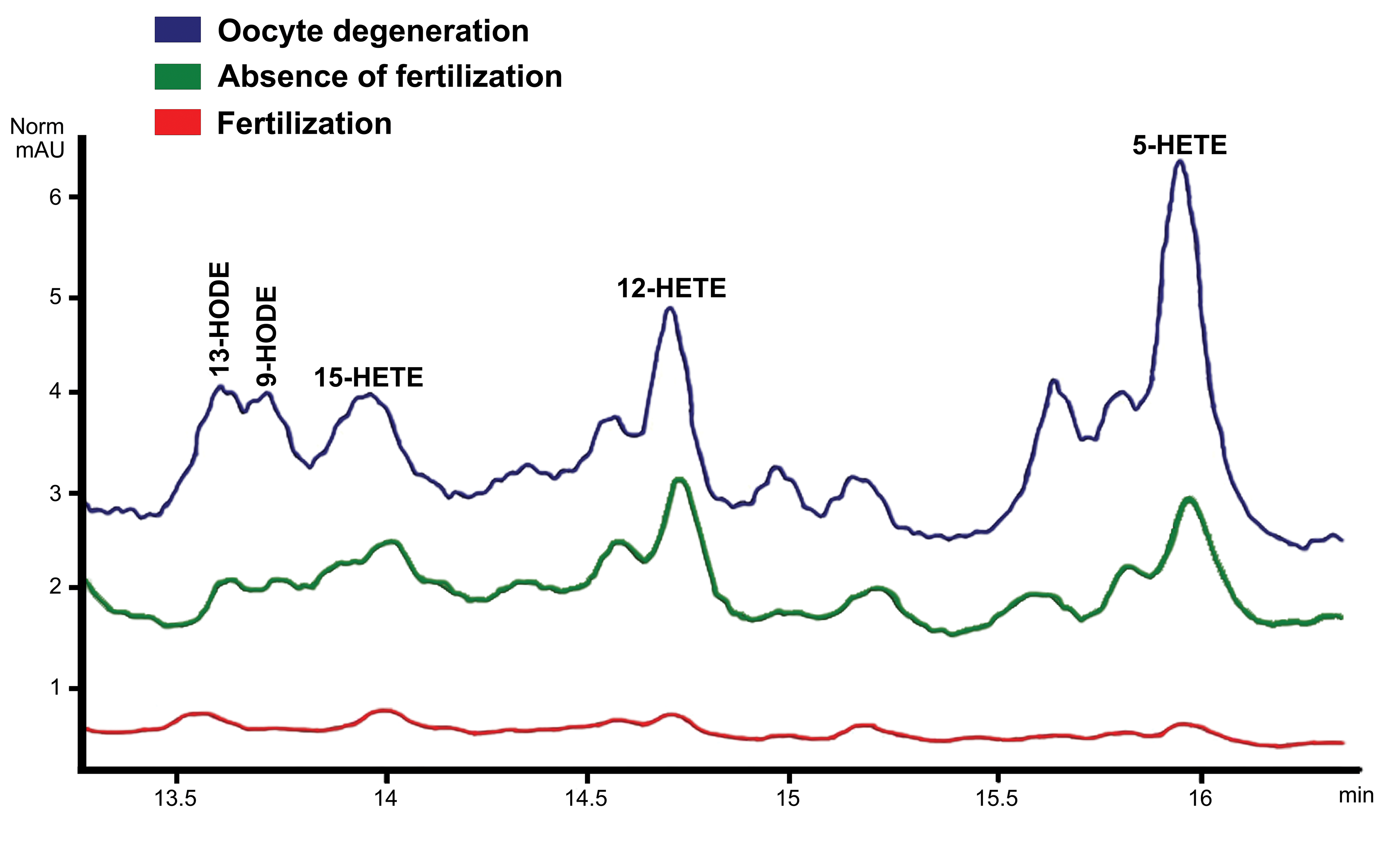

Supplement: S1 Fig — Abbreviations: HETE: hydroxyeicosatetraenoic acid, HODE: hydroxyoctadecadienoic acid, (TIF) [file pone.0119087.s001.tif]

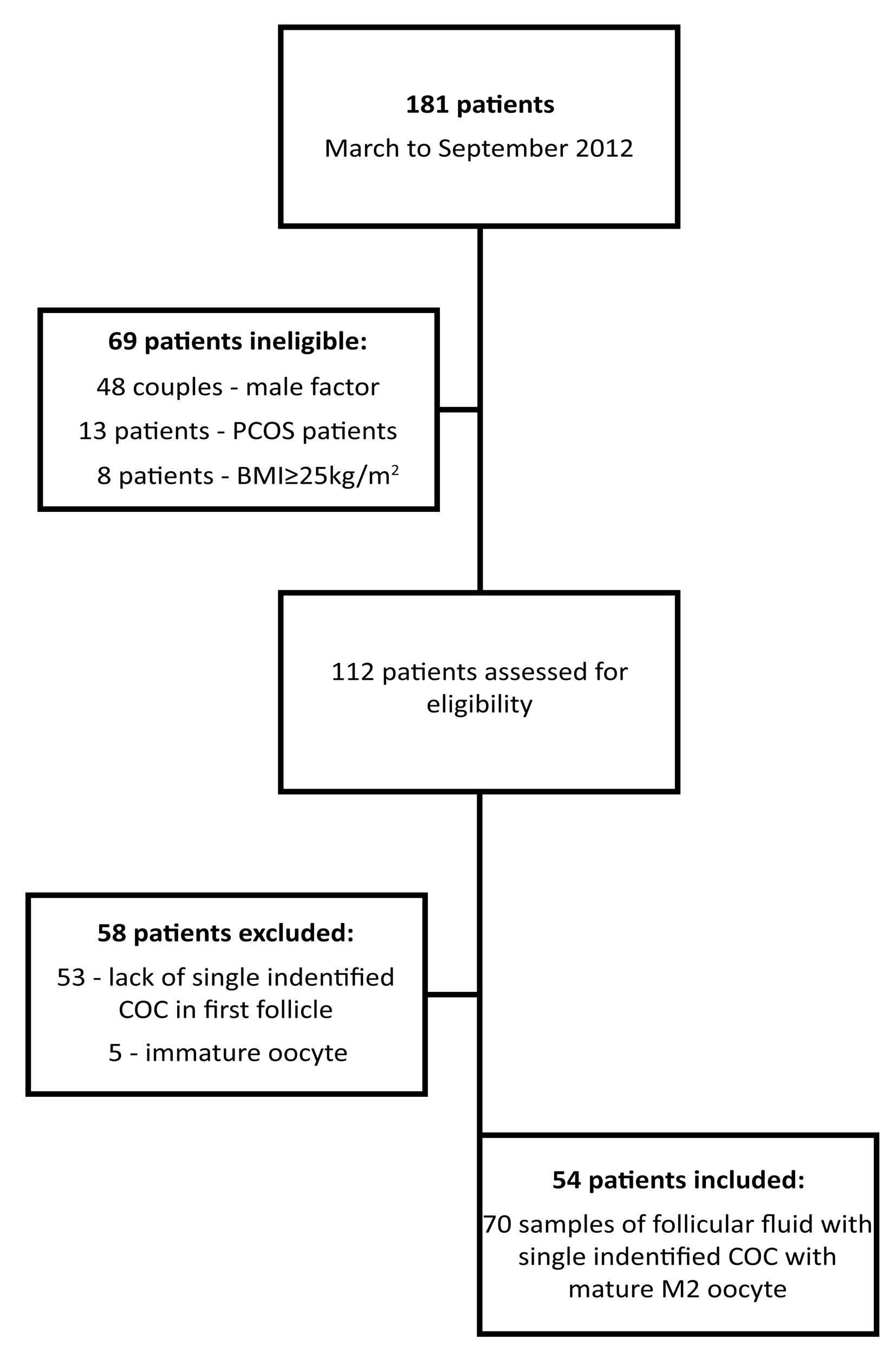

Supplement: S2 Fig — Abbreviations: PCOS: polycystic ovary syndrome; BMI: body mass index; COC: cumulus-oocyte-complex. (TIF) [file pone.0119087.s002.tif]

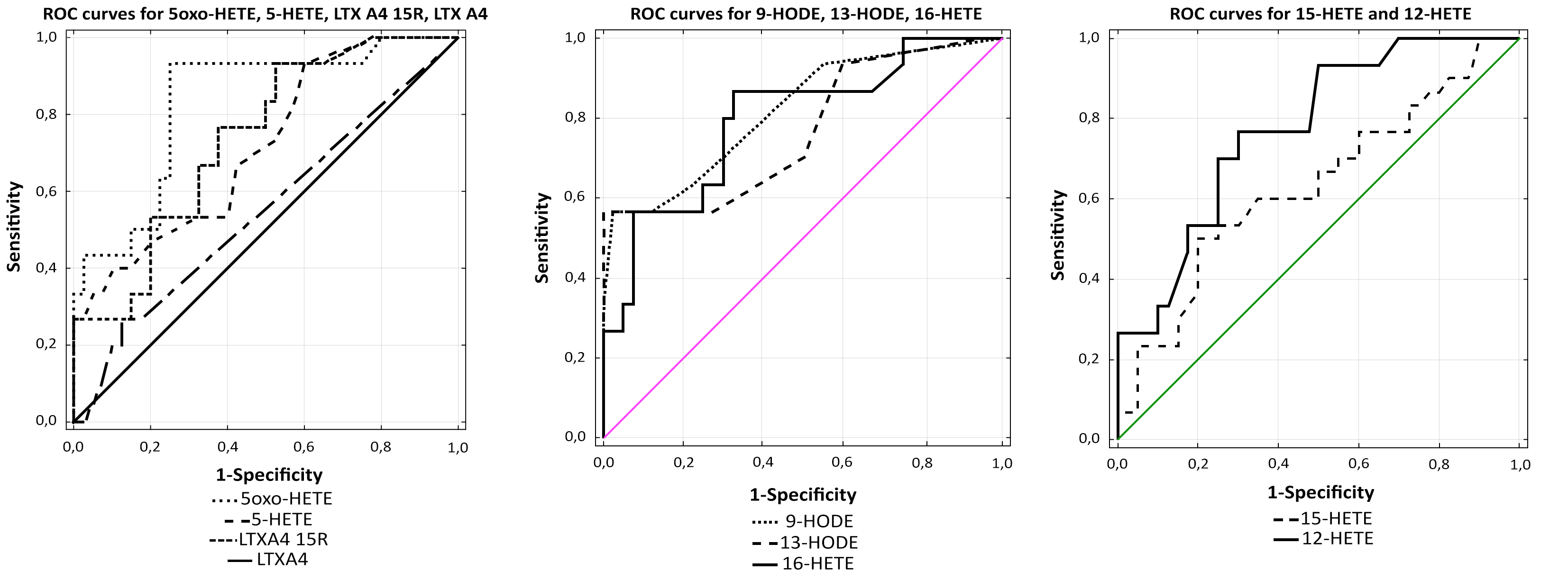

Supplement: S3 Fig — Abbreviations: ROC: receiver operating characteristic, HETE: hydroxyeicosatetraenoic acid; HODE: hydroxyoctadecadienoic acid; LTX: lipoxin. (TIF) [file pone.0119087.s003.tif]
