# Supplementary material for: Arachidonic and Linoleic Acid Derivatives Impact Oocyte ICSI Fertilization – A Prospective Analysis of Follicular Fluid and a Matched Oocyte in a ‘One Follicle – One Retrieved Oocyte – One Resulting Embryo’ Investigational Setting
Source: PLoS One. 2015 Mar 12;10(3):e0119087. doi: 10.1371/journal.pone.0119087 (PMC4357448; doi:10.1371/journal.pone.0119087)
Supplement: S1 Table — Abbreviations: ROC: receiver operating characteristic; HETE: hydroxyeicosatetraenoic acid; HODE: hydroxyoctadecadienoic acid; LTX: lipoxin; LAD: linoleic acid derivatives; AAD: arachidonic acid derivatives; AUC: area under curve. (DOCX) [file pone.0119087.s004.docx]

|  |  | **Cut off point [μg/ml]** | **Sensitivity [%]** | **Specificity [%]** | **AUC** | **95% CI** |
| --- | --- | --- | --- | --- | --- | --- |
| **LAD** | **9-HODE** | 0.002 | 100 | 50 | 0.725 | (0.479-0.971) |
|  | **13-HODE** | 0.005 | 100 | 29 | 0.729 | (0.510-0.949) |
| **AAD** | **5oxo-ETE** | 0.036 | 83 | 71 | 0.816 | (0.622-1.000) |
|  | **16-HETE** | 0.053 | 100 | 33 | 0.681 | (0.361-1.000) |
|  | **LTX A4** | 0.006 | 100 | 0 | 0.286 | (0.125-0.446) |
|  | **LTX A4 15R** | 0.221 | 100 | 50 | 0.943 | (0.870-1.000) |
