# Supplementary material for: Arachidonic and Linoleic Acid Derivatives Impact Oocyte ICSI Fertilization – A Prospective Analysis of Follicular Fluid and a Matched Oocyte in a ‘One Follicle – One Retrieved Oocyte – One Resulting Embryo’ Investigational Setting
Source: PLoS One. 2015 Mar 12;10(3):e0119087. doi: 10.1371/journal.pone.0119087 (PMC4357448; doi:10.1371/journal.pone.0119087)
Supplement: S2 Table — *U Mann Whitney; Abbreviations: LAD: linoleic acid derivatives; AAD: arachidonic acid derivatives; HETE: hydroxyeicosatetraenoic acid; HODE: hydroxyoctadecadienoic acid; LTX: lipoxin; SD: standard deviation. (DOCX) [file pone.0119087.s005.docx]

|  | **[μg/ml]** | **Class A (n=29)**  **Mean ± SD** | **Class B (n=11)**  **Mean ± SD** | **p*** |
| --- | --- | --- | --- | --- |
| **LAD** | **9-HODE** | 0.002 ± 0.002 | 0.002 ± 0.003 | NS |
|  | **13-HODE** | 0.004 ± 0.003 | 0.004 ± 0.004 | NS |
| **AAD** | **5-HETE** | 0.007 ± 0.008 | 0.006 ± 0.006 | NS |
|  | **5oxo-ETE** | 0.054 ± 0.056 | 0.055 ± 0.062 | NS |
|  | **12-HETE** | 0.044 ± 0.043 | 0.054 ± 0.045 | NS |
|  | **15-HETE** | 0.023 ± 0.027 | 0.032 ± 0.034 | NS |
|  | **16-HETE** | 0.037 ± 0.037 | 0.039 ± 0.033 | NS |
|  | **LTX A4** | 0.002 ± 0.003 | 0.002 ± 0.003 | NS |
|  | **LTX A4 15R** | 0.103 ± 0.150 | 0.073 ± 0.097 | NS |
