# Supplementary material for: Arachidonic and Linoleic Acid Derivatives Impact Oocyte ICSI Fertilization – A Prospective Analysis of Follicular Fluid and a Matched Oocyte in a ‘One Follicle – One Retrieved Oocyte – One Resulting Embryo’ Investigational Setting
Source: PLoS One. 2015 Mar 12;10(3):e0119087. doi: 10.1371/journal.pone.0119087 (PMC4357448; doi:10.1371/journal.pone.0119087)
Supplement: S3 Table — *U Mann Whitney; Abbreviations: LAD: linoleic acid derivatives; AAD: arachidonic acid derivatives; HETE: hydroxyeicosatetraenoic acid; HODE: hydroxyoctadecadienoic acid; LTX: lipoxin; SD: standard deviation. (DOCX) [file pone.0119087.s006.docx]

|  | **[μg/ml]** | **Blastocyst stage (n=15)**  **Mean ± SD** | **Development arrest (n=7)**  **Mean ± SD** | **p*** |
| --- | --- | --- | --- | --- |
| **LAD** | **9-HODE** | 0.001 ± 0.000 | 0.002 ± 0.001 | NS |
|  | **13-HODE** | 0.002 ± 0.001 | 0.003 ± 0.002 | NS |
| **AAD** | **5-HETE** | 0.005 ± 0.004 | 0.006 ± 0.003 | NS |
|  | **5oxo-ETE** | 0.050 ± 0.049 | 0.056 ± 0.053 | NS |
|  | **12-HETE** | 0.043 ± 0.027 | 0.048 ± 0.037 | NS |
|  | **15-HETE** | 0.024 ± 0.020 | 0.026 ± 0.025 | NS |
|  | **16-HETE** | 0.039 ± 0.025 | 0.044 ± 0.035 | NS |
|  | **LTX A4** | 0.001 ± 0.003 | 0.002 ± 0.003 | NS |
|  | **LTX A4 15R** | 0.118 ± 0.129 | 0.098 ± 0.139 | NS |
