# Supplementary material for: Arachidonic and Linoleic Acid Derivatives Impact Oocyte ICSI Fertilization – A Prospective Analysis of Follicular Fluid and a Matched Oocyte in a ‘One Follicle – One Retrieved Oocyte – One Resulting Embryo’ Investigational Setting
Source: PLoS One. 2015 Mar 12;10(3):e0119087. doi: 10.1371/journal.pone.0119087 (PMC4357448; doi:10.1371/journal.pone.0119087)
Supplement: S4 Table — *U Mann Whitney; Abbreviations: LAD: linoleic acid derivatives; AAD: arachidonic acid derivatives; HETE: hydroxyeicosatetraenoic acid; HODE: hydroxyoctadecadienoic acid; LTX: lipoxin; SD: standard deviation. (DOCX) [file pone.0119087.s007.docx]

|  | **[μg/ml]** | **Pregnancy (n=9)**  **Mean ± SD** | **No pregnancy (n=9)**  **Mean ± SD** | **p*** |
| --- | --- | --- | --- | --- |
| **LAD** | **9-HODE** | 0.001 ± 0.000 | 0.001 ± 0.000 | NS |
|  | **13-HODE** | 0.002 ± 0.001 | 0.002 ± 0.002 | NS |
| **AAD** | **5-HETE** | 0.005 ± 0.004 | 0.004 ± 0.005 | NS |
|  | **5oxo-ETE** | 0.049 ± 0.051 | 0.056 ± 0.049 | NS |
|  | **12-HETE** | 0.040 ± 0.029 | 0.043 ± 0.027 | NS |
|  | **15-HETE** | 0.023 ± 0.020 | 0.026 ± 0.034 | NS |
|  | **16-HETE** | 0.037 ± 0.039 | 0.038± 0.035 | NS |
|  | **LTX A4** | 0.001 ± 0.003 | 0.001 ± 0.004 | NS |
|  | **LTX A4 15R** | 0.105 ± 0.139 | 0.101 ± 0.089 | NS |
